# Seizures in the respiratory ICU: single-center study of patients with new-onset seizures

**DOI:** 10.1186/cc10913

**Published:** 2012-03-20

**Authors:** D Talwar, V Nair, J Chudiwal

**Affiliations:** 1 Metro Center for Respiratory Diseases, Noida, India

## Introduction

New-onset seizures in the ICU are a diagnostic and management challenge as patients have multiple comorbidities and receive various antibiotics. In the respiratory ICU with different patient profiles, etiopathogenesis of seizures is unreported.

## Methods

We retrospectively analyzed the profile of 3,342 patients admitted to the RICU from 2006 to 2011. A computerized search revealed 79 patients (2.4%) with new-onset seizures. Complete clinical, laboratory, radiological and treatment profiles were recorded and statistically analyzed using the chi-square test, odds ratio and relative risk of individual variable.

## Results

Of 79 patients, 44 patients (55.7%) were males and the mean age was 61.28 ± 19.57 years. Severe sepsis was diagnosed in 32 (40.5%) and multiorgan failure in 19 (24.1%). Head CT done in 65 (82.3%) patients was reported abnormal in 34 (52.3%; *P *= 0.072) patients. Lumbar puncture was done in 40 (50.6%) with five (12.5%) patients having meningitis. Thirteen of 37 (35.1%) patients showed focal activity on EEG (*P *= 0.27; OR = 1.73). Electrolyte abnormalities were: hypermagnesemia in 20 patients (25.3%), hypocalcemia in 17 patients (21.5%), and hypernatremia in 13 patients (16.5%), hyponatremia in three patients (3.8%) and hypomagnesia in four (5.17%) cases. The antibiotics received revealed 27 (34.2%; RR = 1.27) patients on levofloxacin alone or in combination. Twenty-eight of 79 (35.4%) patients were on carbapenems with meropenem in 23/79 (29.1%; RR = 1.21) and imipenem in 5/79 (6.32%; RR = 0.41). See Table 1.

**Table 1:** Attributable causes of seizures in RICU cases (*n *= 79)

Anoxia	8	10.1%
Metabolic	15	19.0%
Drugs only	16	20.3%
CNS infection	5	6.3%
Trauma	2	2.5%
Alcohol	5	6.3%
Multiple	22	27.8%
Miscellaneous	6	7.6%

## Conclusion

New-onset seizure in RICU cases is multifactorial in origin. Use of levofloxacin in combination had the highest relative risk of developing seizure although when given alone the risk is rare (2.1%). Severe sepsis with multiorgan failure being seen in nearly one-half of RICU cases may decrease seizure threshold in these patients.

